# Supplementary material for: A branching model of lineage differentiation underpinning the neurogenic potential of enteric glia
Source: Nat Commun. 2023 Sep 22;14:5904. doi: 10.1038/s41467-023-41492-3 (PMC10516949; doi:10.1038/s41467-023-41492-3)
Supplement: Supplementary file 1 — Supplementary Information [file 41467_2023_41492_MOESM1_ESM.pdf]

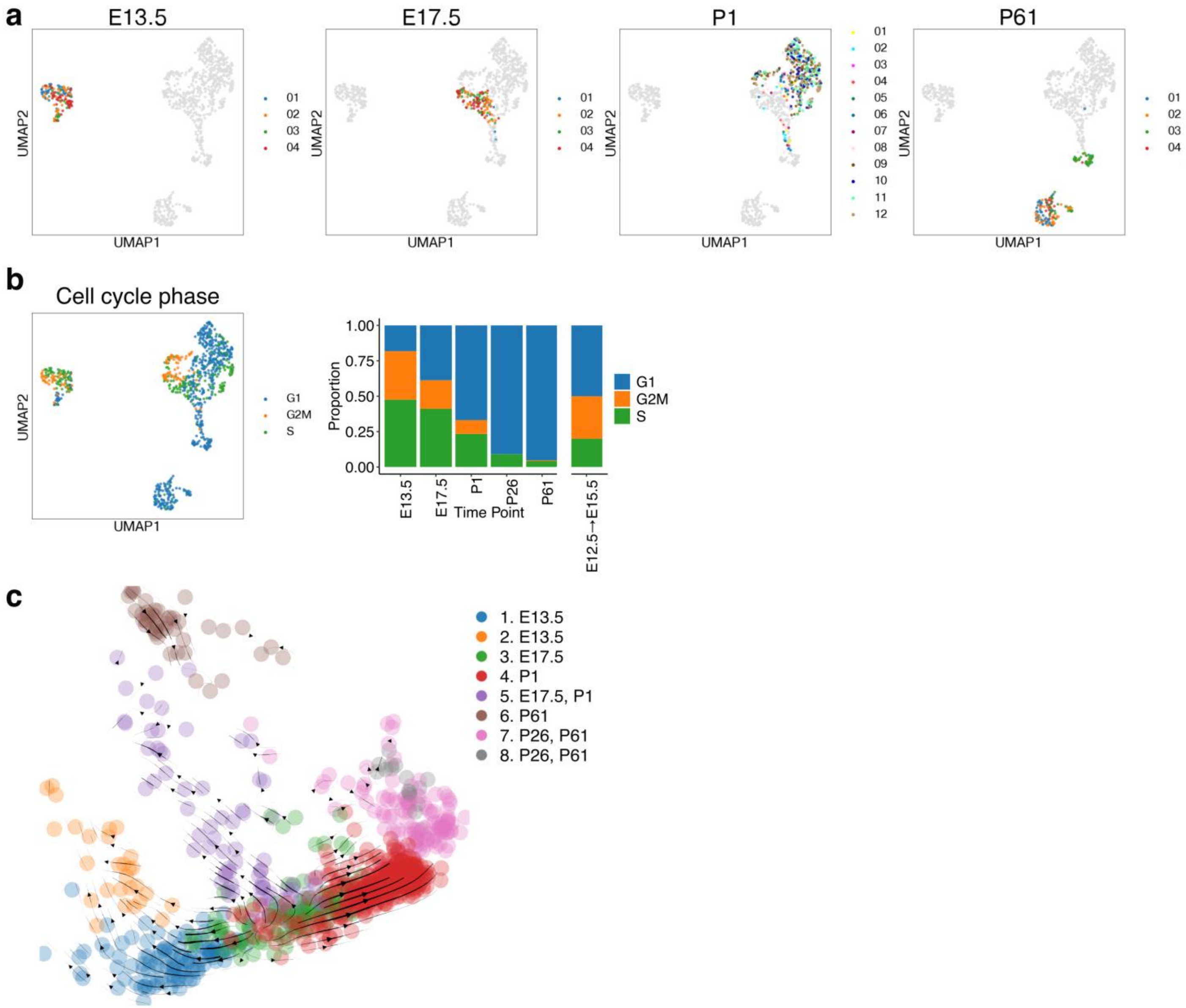

Supplementary Figure 1. Properties of the transcriptional landscape of the developing ENS.

a, UMAP representation of the scRNA-seq dataset (904 transcriptomes) with cells from an individual time point coloured by plate and cells from other time points coloured in grey. Note the lack of batch effects. **b,** UMAP representation of the entire scRNA-seq dataset coloured by inferred cell cycle phase and bar plot showing the proportions of inferred cell cycle phases at each time point. **c,** PCA plot shown with velocity field suggesting the direction of cell differentiation. Source data is provided as a Source Data file.

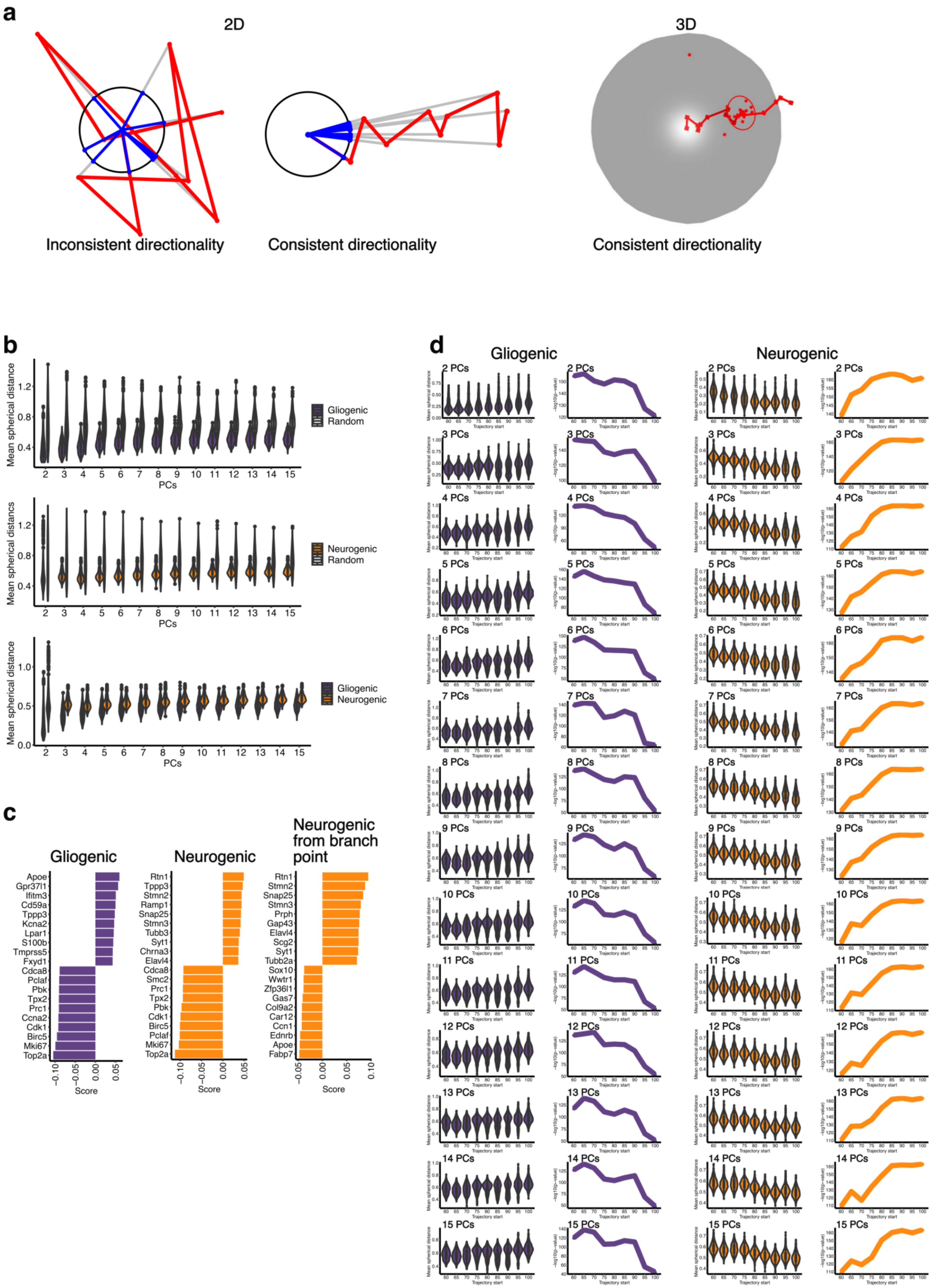

a
2D
3D
Inconsistent directionality
Consistent directionality
Consistent directionality
b
Mean spherical distance
PCs
Gliogenic
Random
Mean spherical distances
Neurogenic
Random
Mean spherical distance
Gliogenic
Neurogenic
c
Gliogenic
Apoe
Gpr37l1
Ifitm3
Cd59a
Tppp3
Kcna2
Lpar1
S100b
Tmprss5
Fxyd1
Cdca8
Pclaf
Pbk
Tpx2
Prc1
Ccna2
Cdk1
Birc5
Mki67
Top2a
Score
Neurogenic
Rtn1
Tppp3
Stmn2
Ramp1
Snap25
Stmn3
Tubb3
Syt1
Chrna3
Elavl4
Cdca8
Smc2
Prc1
Tpx2
Pbk
Cdk1
Birc5
Pclaf
Mki67
Top2a
Score
Neurogenic from branch point
Rtn1
Stmn2
Snap25
Stmn3
Prph
Gap43
Elavl4
Scg2
Syt1
Tubb2a
Sox10
Wwtr1
Zfp36l1
Gas7
Col9a2
Car12
Ccn1
Ednrb
Apoe
Fabp7
Score
d
Gliogenic
Neurogenic
2 PCs
3 PCs
4 PCs
5 PCs
6 PCs
7 PCs
8 PCs
9 PCs
10 PCs
11 PCs
12 PCs
13 PCs
14 PCs
15 PCs
Mean spherical distance
-log10(p-value)
Trajectory start

Supplementary Figure 2. TrajectoryGeometry, a novel tool for the analysis of developmental trajectories.

a, Illustration of the TrajectoryGeometry algorithm. A trajectory in 2 dimensions can be projected onto the 1-sphere (a circle). If this has inconsistent directionality the projections will disperse around the circumference, whereas if the trajectory has a consistent directionality these will be restricted to a small region. This approach can be extended to spheres in an arbitrary number of dimensions e.g. a trajectory in 3 dimensions can be projected onto the 2-sphere. Here the closeness of projections on the surface of the sphere can be described by a circle that shows the mean spherical distance from the centre of the projections. **b,** Violin plots showing the mean spherical distance (radii of the circles as shown in **a**) for 1000 paths sampled from glial and neuronal trajectories in comparison to 1000 random trajectories, and directly comparing glial and neuronal trajectories, when considering different numbers of PCs (accompanying statistics included in Supplementary tables 1-3). **c,** Bar plots showing the top 10 TrajectoryGeometry genes positively associated (positive score) and top 10 TrajectoryGeometry genes negatively associated (negative score) with the directionality of the indicated trajectories. **d,** Violin plots and line graphs showing –log10(p-value) for the significance of neuronal and glial trajectory directionality (1000 sampled paths), relative to 1000 random trajectories, starting from successively later points in pseudotime, as the branch point is approached. In **b** and **d** the box centre represents the median, lower and upper hinges correspond to the first and third quartiles (the 25th and 75th percentiles). The whiskers extend from the hinge to the largest value no further than 1.5 * inter-quartile from the hinge. Points beyond the end of these are plotted individually. Source data are provided as Source Data files.

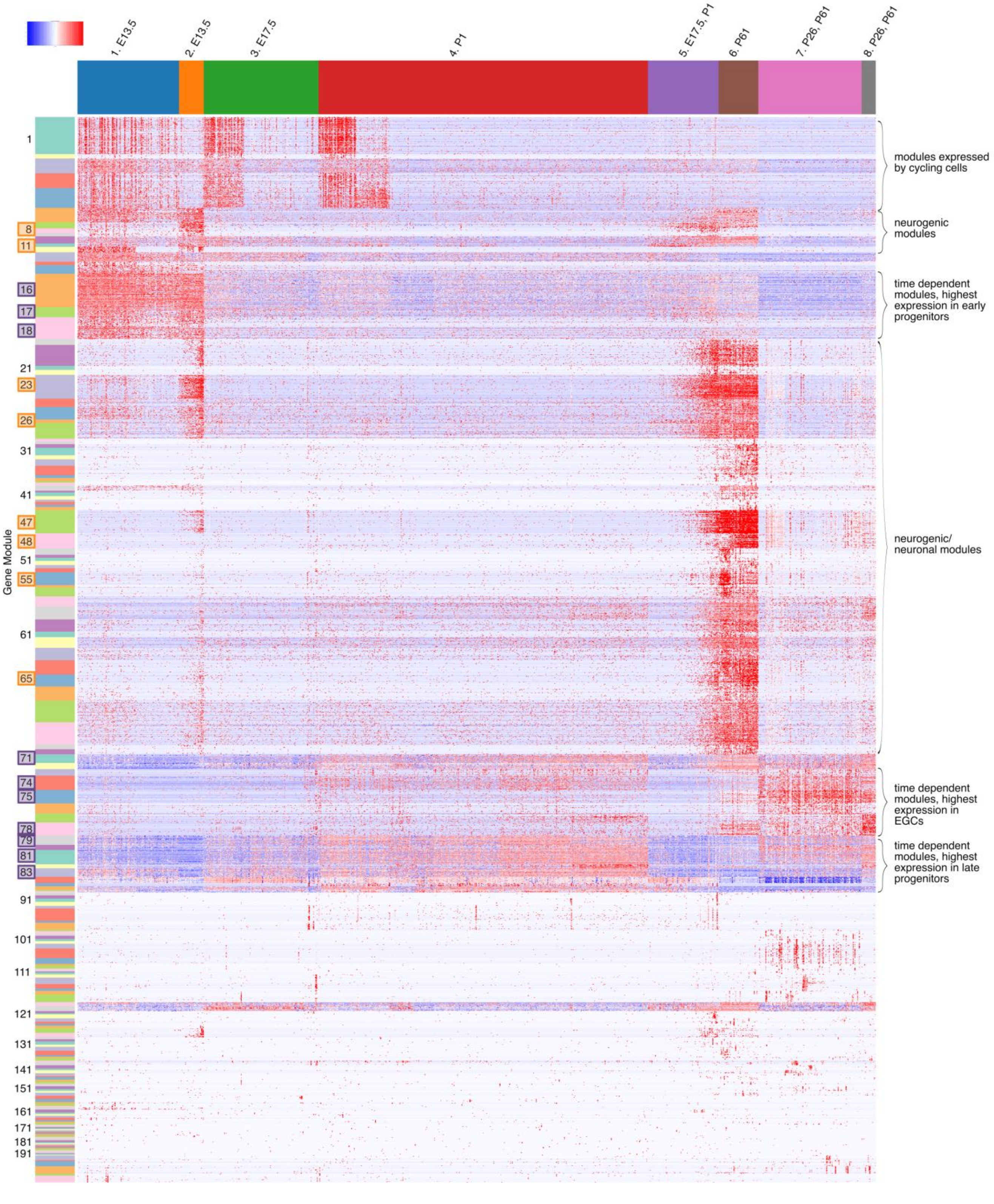
1. E13.5
2. E13.5
3. E17.5
4. P1
5. E17.5, P1
6. P61
7. P26, P61
8. P26, P61
Gene Module
1
8
11
16
17
18
21
23
26
31
41
47
48
51
55
61
65
71
74
75
78
79
81
83
91
101
111
121
131
141
151
161
171
181
191
modules expressed by cycling cells
neurogenic modules
time dependent modules, highest expression in early progenitors
neurogenic/ neuronal modules
time dependent modules, highest expression in EGCs
time dependent modules, highest expression in late progenitors

Supplementary Figure 3. ANTLER gene modules.

Heatmap (scaled normalised expression) of all gene modules (numbered on the left) identified using the ANTLER algorithm. Orange boxes around module numbers refer to neurogenic modules described in the main text; purple boxes around module numbers refer to modules with time-dependent expression described in the main text. Cell clusters are shown at the top.

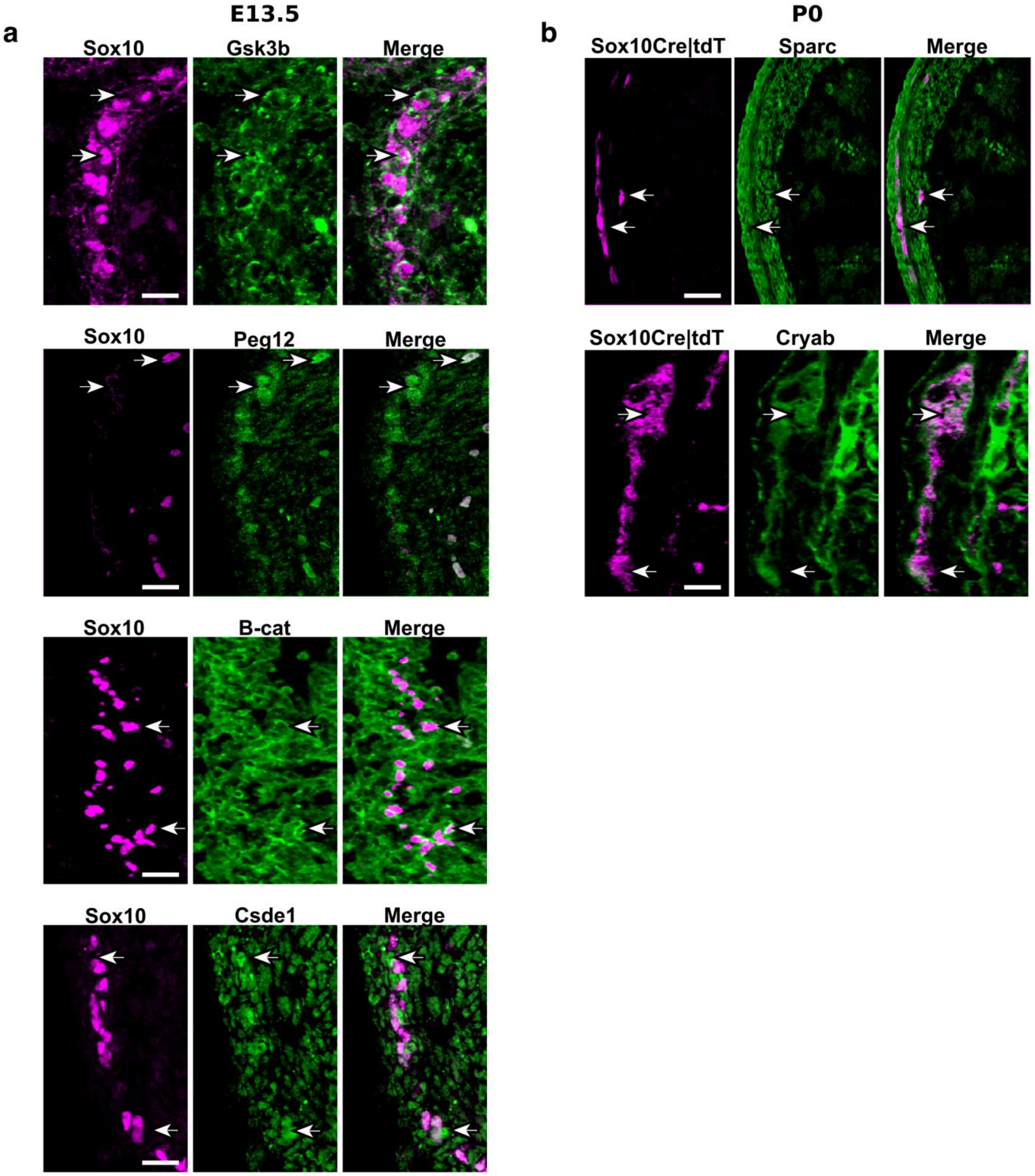
E13.5
a
Sox10
Gsk3b
Merge
Sox10
Peg12
Merge
Sox10
B-cat
Merge
Sox10
Csde1
Merge
P0
b
Sox10Cre|tdT
Sparc
Merge
Sox10Cre|tdT
Cryab
Merge

Supplementary Figure 4. Validation of gene expression in the developing ENS.

Immunostaining for the validation of expression of genes from ANTLER GMs in the ENS of mice at E13.5 (**a**) and P0 (**b**). Arrows point to cells of the ENS lineages in the muscularis externa of the gut, as identified by immunostaining for the transcription factor SOX10 (**a**) and tdT from the transgenic reporter Sox10Cre|tdT (**b**). Scale bars: 50 µm. Immunostainings were performed twice independently with similar results.

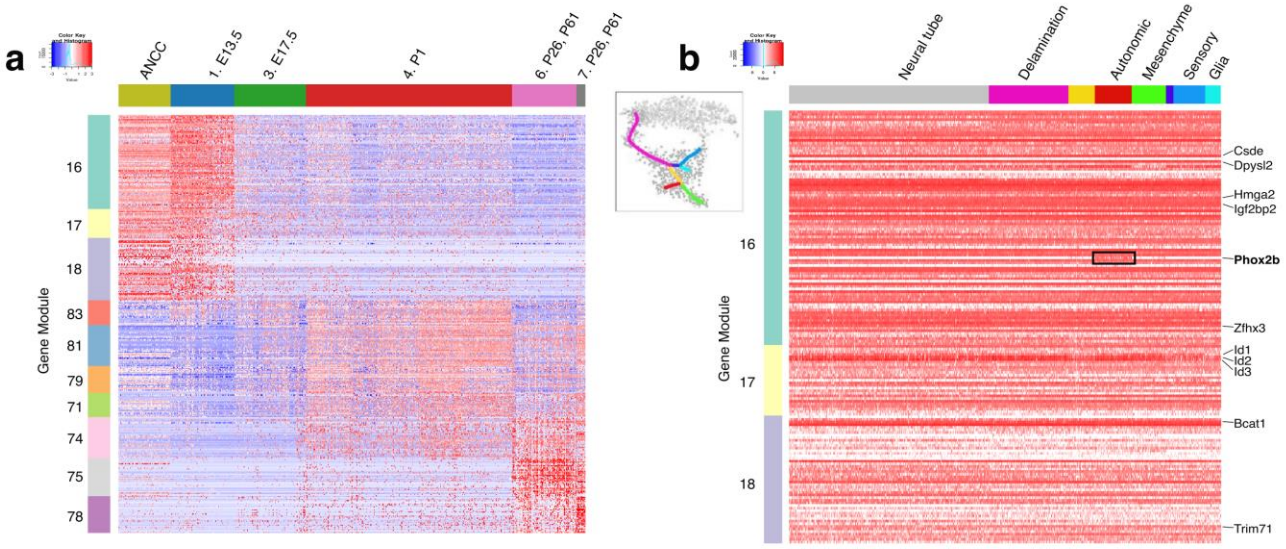

Supplementary Figure 5. Neurogenic GM expression by early neural crest and ANCCs.

a, Heatmap (scaled normalised expression) of the indicated GMs (left) in ANCCs and the tdT^+ ENS cell clusters (top). **b,** Heatmap of gene expression (normalised, not scaled) for the neurogenic GM16-18 (left) in the indicated neural crest cell populations. *Phox2b* expression is restricted to the autonomic lineage.

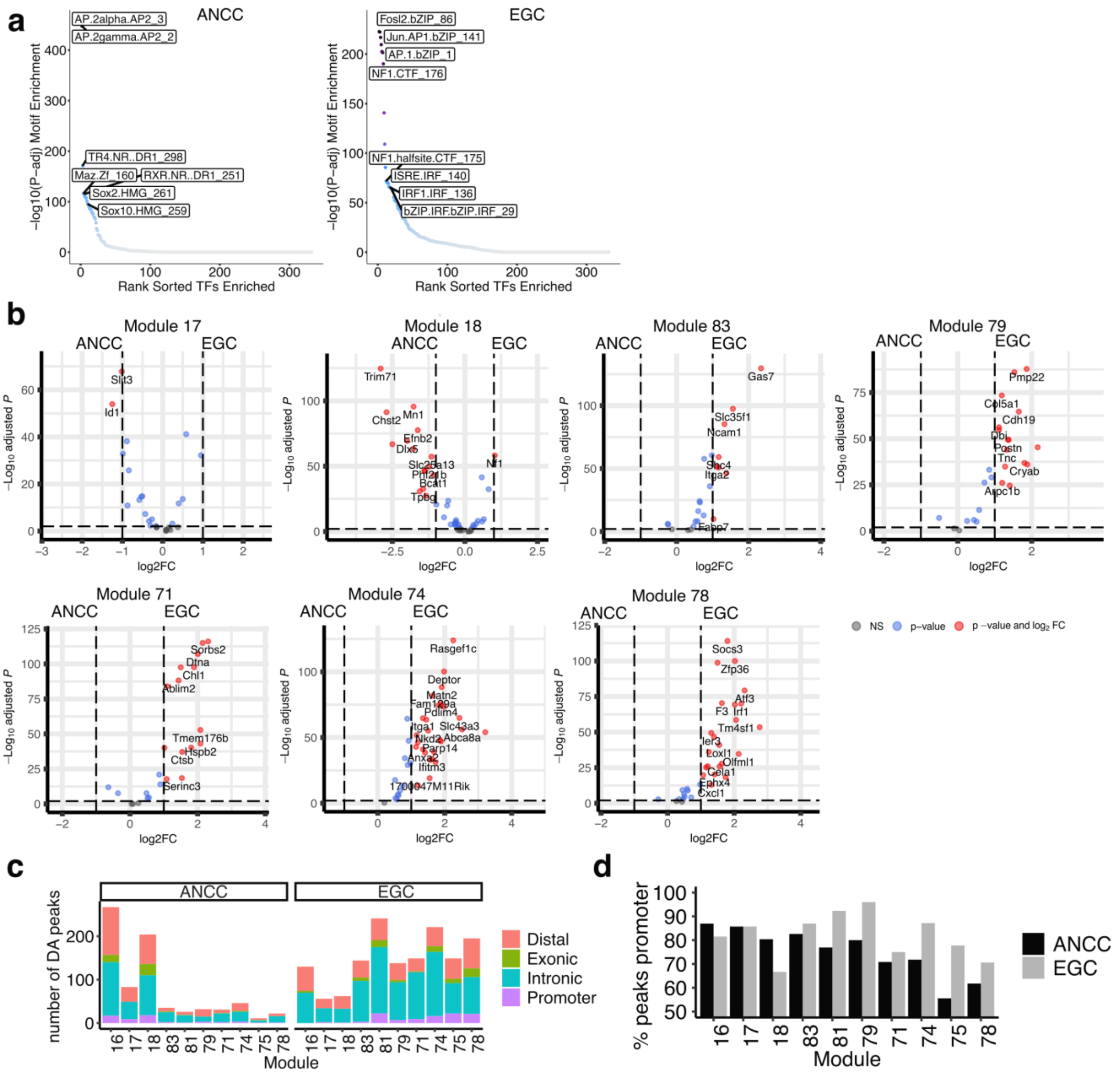

Supplementary Figure 6. Chromatin accessibility in ANCCs and EGCs.

a, Plot showing enrichment of HOMER motifs in DA peaks (log2FC > 1 & padj < 0.01) in ANCCs and EGCs. **b,** Volcano plots showing differential accessibility between ANCCs and EGCs (based on gene score) for genes from the indicated GMs. **c,** Bar plot showing the number of DA peaks (log2FC > 1 & padj < 0.01) in ANCCs and EGCs. Results shown for peaks associated with genes from the indicated GMs. Colour indicates the gene region quantified (promoter, exonic, intronic, distal). **d,** Bar plot showing the percentage of genes in the indicated GMs that have at least one peak in their promoter region. Source data are provided as Source Data files.

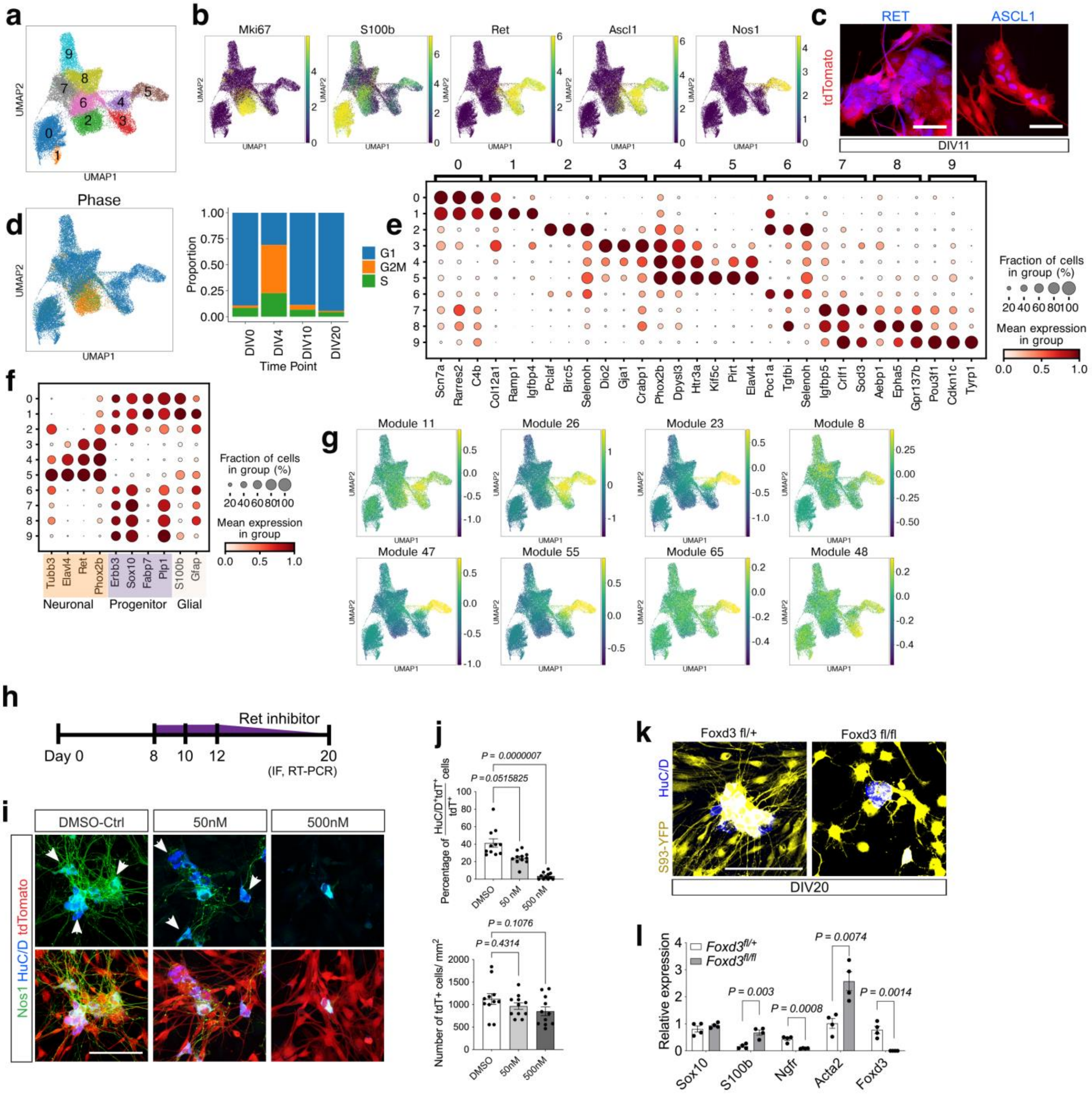

a
b
Mki67
S100b
Ret
Ascl1
Nos1
c
RET
ASCL1
tdTomato
DIV11
d
Phase
Proportion
Time Point
DIV0
DIV4
DIV10
DIV20
G1
G2M
S
e
Fraction of cells in group (%)
Mean expression in group
f
Neuronal
Progenitor
Glial
g
Module 11
Module 26
Module 23
Module 8
Module 47
Module 55
Module 65
Module 48
h
Ret inhibitor
Day 0
(IF, RT-PCR)
i
DMSO-Ctrl
50nM
500nM
Nos1 HuC/D tdTomato
j
P = 0.0000007
P = 0.0515825
P = 0.1076
P = 0.4314
Number of tdT+ cells/ mm2
k
Foxd3 fl/+
Foxd3 fl/fl
HuC/D
S93-YFP
DIV20
l
Foxd3fl/+
Foxd3fl/fl
Relative expression
P = 0.003
P = 0.0008
P = 0.0074
P = 0.0014
Sox10
S100b
Ngfr
Acta2
Foxd3

Supplementary Figure 7. Enteric glial cell cultures recapitulate in vivo neurogenesis.

a, UMAP representation of scRNA-seq data (27726 transcriptomes) for ganglioid cultures coloured by Louvain clustering. **b,** Expression of the indicated genes on the UMAP in **a**. **c,** Ganglioid cultures (DIV11) immunostained for RET and ASCL1. The experiment was performed once. **d,** UMAP shown in **a** coloured by inferred cell cycle phase and bar plot showing the proportions of inferred cell cycle phases at each time point in ganglioid cultures. **e,** Dot plot showing the expression of identified marker genes in the clusters shown in **a**. Dot size indicates the fraction of cells expressing an indicated marker while colour indicates mean expression of the gene marker. **f,** Dot plot showing the expression of neuronal, progenitor and glial markers in the clusters shown in **a**. **g,**Expression (score) of selected neurogenesis associated gene modules shown on the UMAP as in **a**. **h,** Experimental strategy for the pharmacological inhibition of RET signalling in ganglioid cultures. **i,** Ganglioid cultures (DIV20) immunostained for nNOS1 and HuC/D after inhibition of RET signalling. tdTomato (red) indicates cells originating from tdT^{+} EGCs. Arrows indicate neurons. **j,** Quantification of HuC/D+ cells upon RET signalling inhibition. Data are mean ± s.e.m. (n=11, field of view per group). Kruskal-Wallis test with Dunn's multiple comparison test (top) and One-way ANOVA with Dunnett's multiple comparison test (bottom). **k,** Representative images of ganglioid cultures derived from lineage-traced (YFP+, yellow) cells from *Sox10CreER*^{T2};*Foxd3*$^{fl/+}$ and *Sox10CreER*^{T2};*Foxd3*$^{fl/fl}$ mice. **l,** Relative gene expression of indicated genes in ganglioid cultures (DIV6) from *Sox10CreER*^{T2};*Foxd3*$^{fl/+}$ and *Sox10CreER*^{T2};*Foxd3*$^{fl/fl}$ mice. Data are mean ± s.e.m. (n = 4). Unpaired two-tailed t-test. Scale bars: 50 µm (c), 100 µm (i, k). Source data are provided as Source Data files.

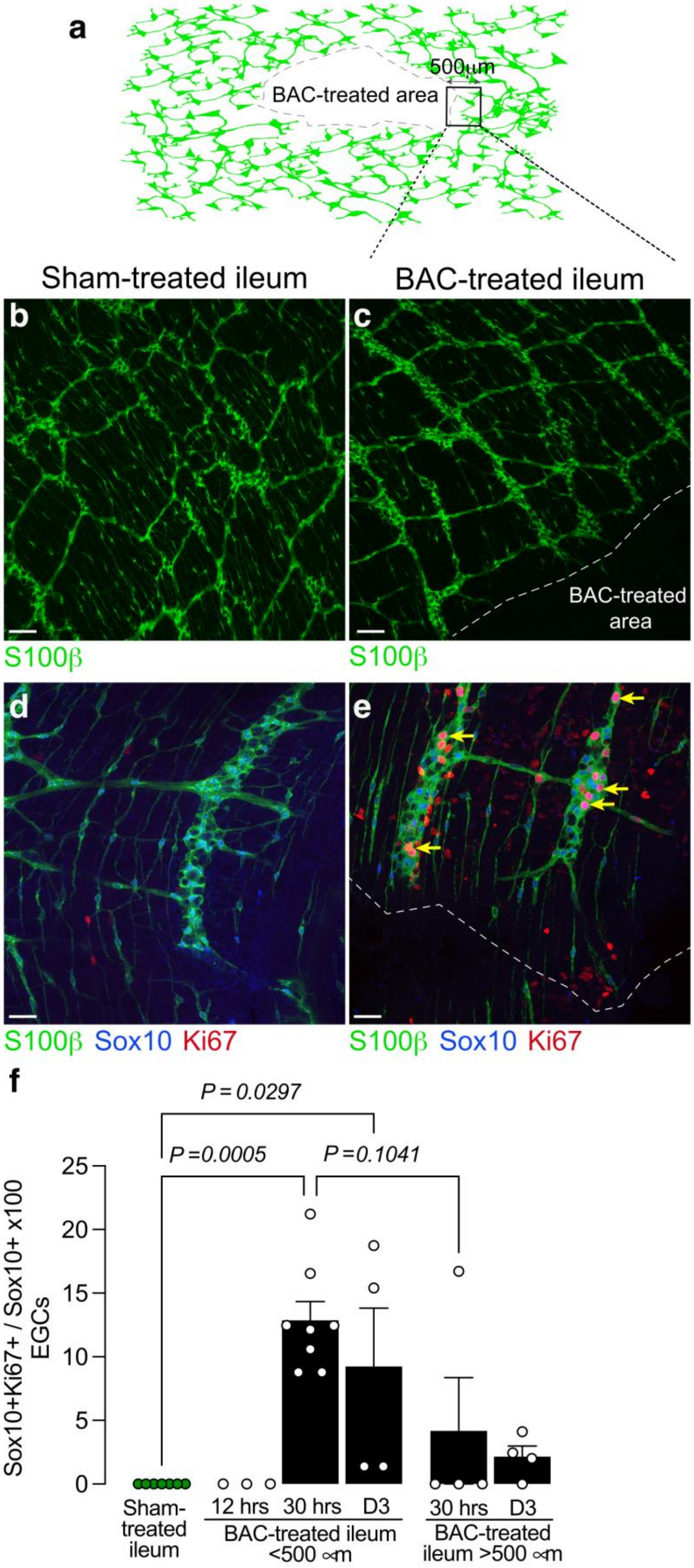
a
500μm
BAC-treated area
Sham-treated ileum
BAC-treated ileum
b
c
BAC-treated area
S100β
S100β
d
e
S100β Sox10 Ki67
S100β Sox10 Ki67
f
P = 0.0297
P = 0.0005
P = 0.1041
Sox10+Ki67+ / Sox10+ x100 EGCs
25
20
15
10
5
0
Sham-treated ileum
12 hrs
30 hrs
D3
BAC-treated ileum <500 ∝m
30 hrs
D3
BAC-treated ileum >500 ∝m

Supplementary Figure 8. BAC-treatment leads to localised loss of ganglia and proliferative activation of enteric glia.

a, Schematic representation of the EGC network of the myenteric plexus within the muscularis externa. BAC treatment leads to localised loss of ganglia (enclosed by the dotted line). Open box indicates the border between ablated and normal plexus and corresponds to the areas presented in panels **c** and **e**. **b, c,** Representative images of myenteric plexus preparations from the ileum of sham- (**b**) and BAC-treated (**c**) mice immunostained for S100β. Dotted line in **c** indicates the border between ablated and normal plexus. Scale bars: 100mm. **d, e,** Higher magnification images of enteric ganglia from sham-treated ileum (**d**) or from the border area of BAC-treated ileum (**e**) immunostained for S100β and Ki67. Ki67+ EGCs (arrows) were detected only in ganglia from BAC-treated gut. Scale bars: 45mm. **f**, Quantification of $SOX10^{+}Ki67^{+}$ EGCs in sham and BAC-treated mice at different hours or days (D) after application of BAC and at <500 µm or >500 µm from the treatment area. n = 7 (sham), n = 3 (12 h), n = 7 (30 h <500 µm), n = 4 (D3 <500 µm, 30 h >500 µm, D3 >500 µm). Kruskal-Wallis test. Data are mean ± s.e.m. Source data is provided as a Source Data file.
