# Supplementary material for: A branching model of lineage differentiation underpinning the neurogenic potential of enteric glia
Source: Nat Commun. 2023 Sep 22;14:5904. doi: 10.1038/s41467-023-41492-3 (PMC10516949; doi:10.1038/s41467-023-41492-3)
Supplement: Supplementary file 3 — Description of Additional Supplementary Files [file 41467_2023_41492_MOESM3_ESM.pdf]

Description of Additional Supplementary Files

Supplementary data 1 – Statistics for the comparison of the glial and random trajectories
Results of two-sided Wilcoxon signed-rank test comparing mean spherical distances for 1000 paths sampled from the glial trajectory in comparison to 1000 random trajectories.

Supplementary data 2 – Statistics for the comparison of the neuronal and random trajectories.
Results of two-sided Wilcoxon signed-rank test comparing mean spherical distances for 1000 paths sampled from the neuronal trajectory in comparison to 1000 random trajectories.

Supplementary data 3 – Statistics for the comparison of the neuronal and glial trajectories
Results of two-sided Mann-Whitney U test comparing mean spherical distances for 1000 paths sampled from the neuronal trajectory in comparison to 1000 paths sampled from the glial trajectory.

Supplementary data 4 – Gene modules
Gene modules identified using the Antler algorithm (https://github.com/juliendelile/Antler).

Supplementary data 5 – Csde1 binding sites
Csde1 binding sites identified using a script published by Moore et al., 2018.

Supplementary data 6 – Csde1 binding site statistics
Results of one-sided permutation testing to assess the enrichment of gene modules in Csde1 binding sites.

Supplementary data 7 – Csde1 knockdown statistics
Results of two-sided Fisher exact test to determine whether gene modules are biased towards genes which are up- or down-regulated upon Csde1 knockdown. Data on CSDE1 knockdown in hESCs taken from Ju Lee et al., 2017.

Supplementary data 8 – Csde1 iCLIP statistics
Results of one-sided hypergeometric test to determine whether gene modules are enriched in RNA targets of Csde1. Data on Csde1 targets taken from Wurth et al., 2016 (iCLIP-seq data in human melanoma).

Supplementary data 9 – ANCC motifs
Motifs enriched in peaks more accessible (logFC >= 1, padj <= 0.01) in ANCCs (one-sided hypergeometric test).

Supplementary data 10 – EGC motifs
Motifs enriched in peaks more accessible (logFC >= 1, padj <= 0.01) in EGCs (one-sided hypergeometric test).

Supplementary data 11 – Contingency table

Contingency table used for testing whether gene modules are biased towards genes which are up- or down-regulated upon Csde1 knockdown (Fisher exact test).
