# Supplementary material for: A branching model of lineage differentiation underpinning the neurogenic potential of enteric glia
Source: Nat Commun. 2023 Sep 22;14:5904. doi: 10.1038/s41467-023-41492-3 (PMC10516949; doi:10.1038/s41467-023-41492-3)
Supplement: Supplementary file 5 — Reporting Summary [file 41467_2023_41492_MOESM5_ESM.pdf]

nature portfolio

Corresponding author(s): Vassilis Pachnis

Last updated by author(s): Aug 23, 2023

Reporting Summary

Nature Portfolio wishes to improve the reproducibility of the work that we publish. This form provides structure for consistency and transparency in reporting. For further information on Nature Portfolio policies, see our Editorial Policies and the Editorial Policy Checklist.

Statistics

For all statistical analyses, confirm that the following items are present in the figure legend, table legend, main text, or Methods section.

n/a	Confirmed	
☐	☒	The exact sample size (*n*) for each experimental group/condition, given as a discrete number and unit of measurement
☐	☒	A statement on whether measurements were taken from distinct samples or whether the same sample was measured repeatedly
☐	☒	The statistical test(s) used AND whether they are one- or two-sided<br>*Only common tests should be described solely by name; describe more complex techniques in the Methods section.*
☒	☐	A description of all covariates tested
☐	☒	A description of any assumptions or corrections, such as tests of normality and adjustment for multiple comparisons
☐	☒	A full description of the statistical parameters including central tendency (e.g. means) or other basic estimates (e.g. regression coefficient) AND variation (e.g. standard deviation) or associated estimates of uncertainty (e.g. confidence intervals)
☐	☒	For null hypothesis testing, the test statistic (e.g. *F*, *t*, *r*) with confidence intervals, effect sizes, degrees of freedom and *P* value noted<br>*Give P values as exact values whenever suitable.*
☒	☐	For Bayesian analysis, information on the choice of priors and Markov chain Monte Carlo settings
☒	☐	For hierarchical and complex designs, identification of the appropriate level for tests and full reporting of outcomes
☒	☐	Estimates of effect sizes (e.g. Cohen's *d*, Pearson's *r*), indicating how they were calculated

Our web collection on statistics for biologists contains articles on many of the points above.

Software and code

Policy information about availability of computer code

Data collection	Olympus FV3000, BDFacsDIVA V8, LAS AF 2.7.3.9723 software, FV31S-SW software (Olympus), Micromanager (2.0), PClamp 10.3
Data analysis	Tophat2, velocyto, RSEM version 1.3.0, STAR aligner version 2.5.2, Trim Galore! (version 0.4.2)<br><br>FASTQC version 0.11.5, RNA-SeQC version 1.1.8, nextflow atacseq pipeline version 1.0dev, 10x Genomics Cell Ranger 3.0.2, 10x Genomics Cell Ranger ATAC 1.2.0, MACS2<br><br>Python version 3<br>Python packages: SCANPY, gprofiler, scVelo, Scrublet<br><br>R versions 3.6.1 and 4.0.0<br>R packages: Antler (https://github.com/juliendelile/Antler), Slingshot, Monocle 2.12.0, ArchR, Seurat version 3, Seurat version 4, EnhancedVolcano, MAST, DESeq2, ggplot2, gplots<br><br>GraphPad Prism 9.0, Fiji/ Image J [Wayne Rasband, NIH], Benchling (2019), Adobe Photoshop 2020, Adobe Illustrator 25.3.1, Inkscape 1.1.2, BioRender.com<br><br>The R Bioconductor package TrajectoryGeometry has been developed as part of this paper and is available at http://bioconductor.org/packages/release/bioc/html/TrajectoryGeometry.html and at https://github.com/AnnaLaddach/TrajectoryGeometry under an MIT License.

Other analysis code is available at https://github.com/AnnaLaddach/ABranchingModelOfENSDevelopment under an MIT License.

For manuscripts utilizing custom algorithms or software that are central to the research but not yet described in published literature, software must be made available to editors and reviewers. We strongly encourage code deposition in a community repository (e.g. GitHub). See the Nature Portfolio guidelines for submitting code & software for further information.

Data

Policy information about availability of data

All manuscripts must include a data availability statement. This statement should provide the following information, where applicable:

- Accession codes, unique identifiers, or web links for publicly available datasets
- A description of any restrictions on data availability
- For clinical datasets or third party data, please ensure that the statement adheres to our policy

Raw and processed scRNA-seq data generated in this manuscript are available on the Gene Expression Omnibus (GEO) under the following accessions: GSE237713 (fluidigm scRNA-seq from small intestine SOX10+ cells), GSE237970 (10X scRNA-seq from ganglioid cultures), GSE160196 (bulk RNA-seq from ganglioid cultures), GSE239305 (scATAC-seq data from EGCs), GSE241522 (bulk ATAC-seq data from ganglioid cultures), GSE240190 (scATAC-seq data from trunk/vagal neural crest cells; specific sample GSM7687949), GSE240636 (bulk RNA-seq of EGCs from BAC-treated mice). The following previously generated data used in this manuscript is available on GEO: GSE182715 (fluidigm scRNA-seq from small intestine P60 enteric nervous system cells), GSE129114 (fluidigm scRNA-seq from vagal/trunk neural crest). The previously generated fluidigm scRNA-seq data from small intestine SOX10+ cells is available at www.ebi.ac.uk/arrayexpress/experiments/E-MTAB-5553. scATAC-seq fresh cortical adult mouse brain (P50) data was downloaded from 10X (https://www.10xgenomics.com/resources/datasets?menu%5Bproducts.name%5D=Single%20Cell%20ATAC&page=1&configure%5Bfacets%5D%5B0%5D=chemistryVersionAndThroughput&configure%5Bfacets%5D%5B1%5D=pipeline.version&configure%5BhitsPerPage%5D=500). Adult mouse cortical scRNA-seq data from the Allen Institute for Brain Science was downloaded from https://www.dropbox.com/s/kqsy9tvsklbu7c4/allen_brain.rds. Gene lists used for cell cycle scoring were obtained from Tirosh et al., 2015. iCLIP-seq data, which identified direct RNA targets of human CSDE1 in melanoma cells were obtained from Wurth et al., 2016. RNA-seq data on genes differentially expressed in hESCs after CSDE1 knockdown was obtained from Ju Lee et al., 2017. Human-mouse gene mappings were obtained from the NCBI HomoloGene database (ncbi.nlm.nih.gov/homologene). The GRCm38 genome is available from Ensembl.

Research involving human participants, their data, or biological material

Policy information about studies with human participants or human data. See also policy information about sex, gender (identity/presentation), and sexual orientation and race, ethnicity and racism.

Reporting on sex and gender	n/a
Reporting on race, ethnicity, or other socially relevant groupings	n/a
Population characteristics	n/a
Recruitment	n/a
Ethics oversight	n/a

Note that full information on the approval of the study protocol must also be provided in the manuscript.

Field-specific reporting

Please select the one below that is the best fit for your research. If you are not sure, read the appropriate sections before making your selection.

[x] Life sciences [ ] Behavioural & social sciences [ ] Ecological, evolutionary & environmental sciences

For a reference copy of the document with all sections, see nature.com/documents/nr-reporting-summary-flat.pdf

Life sciences study design

All studies must disclose on these points even when the disclosure is negative.

Sample size	Sample size was determined based on preliminary experiments or reports in the literature. No statistical analysis was carried out prior to experiments to determine the size of experimental groups, but sample sizes were large enough to determine the effect size.
Data exclusions	No data was excluded
Replication	Experiments were repeated with at least two biologically independent experiments.
Randomization	Littermates (of mixed sex) were randomly assigned to experimental groups in an age range of 2-4 months.
Blinding	Blinding was performed wherever possible.

Reporting for specific materials, systems and methods

We require information from authors about some types of materials, experimental systems and methods used in many studies. Here, indicate whether each material, system or method listed is relevant to your study. If you are not sure if a list item applies to your research, read the appropriate section before selecting a response.

Materials & experimental systems

n/a	Involved in the study
☐	☒ Antibodies
☐	☒ Eukaryotic cell lines
☒	☐ Palaeontology and archaeology
☐	☒ Animals and other organisms
☒	☐ Clinical data
☒	☐ Dual use research of concern
☒	☐ Plants

Methods

n/a	Involved in the study
☒	☐ ChIP-seq
☒	☐ Flow cytometry
☒	☐ MRI-based neuroimaging

Antibodies

Antibodies used

APOE (1:200, Abcam, ab1906)
β-CATENIN (1:300, BD Transduction Labs, 610154)
CALB1 (1:500, Chemicon, AB1778)
CRYAB (1:250, Abcam, ab13496)
CSDE1 (1:200, Abcam, ab201688)
DBI (1:200, Aviva Systems Biology, ARP33135_P050)
DPYSL2 (1:300, Proteintech, 14686)
FCGRT (1:300, Thermo Fisher Scientific, PA5-42871)
GFP (1:500, Nacalai Tesque, 04404-84)
GFP (1:400, Abcam, ab13970)
GFAP (1:500, Abcam, ab4674)
GFK3β (1:200, Abcam, ab32391)
HMGA2 (1:300, Abcam, ab97276)
HuC/D (1:400, Thermo Fisher Scientific, A-21271)
IGF2BP2 (1:300, Abcam, ab124930)
Ki67 (1:200, BD Biosciences, 550609)
nNOS1 (1:300, Abcam, ab1376)
NPY (1:400, Biogenesis, 6730-0004)
PEG12 (1:200, RayBiotech, NP_038816.1)
PHOX2B (1:250, R&D Systems, AD4940)
pH3 (1:500, Millipore, 06-570)
S100B (1:500, DAKO, z0311)
SOX10 (1:200, Proteintech, 10422-1-AP)
SPARC (1:400, R&D Systems, AF942)
SYN1 (1:400, Abcam, ab64581)
TuJ1 (1:1000, Biolegend, 801202)
VIP (1:400, Immunostar, 20077)

donkey anti-chicken Alexa Fluor (AF) 488 (1:500, Jackson Immuno Research, 703-545-155)
donkey anti-goat AF 647 (1:500, Invitrogen, A21447)
donkey anti-mouse AF 568 (1:500, Invitrogen, A10037)
donkey anti-rabbit AF 488 (1:500, Invitrogen, A21206)
donkey anti-rat AF 488 (1:500, Invitrogen, A21208)
donkey anti-rabbit AF 405 (1:500, Invitrogen, A48258)

Validation

All antibodies used in our study are commercially available and have been validated by the manufacturer. The catalog number of each antibody is described above.

Eukaryotic cell lines

Policy information about cell lines and Sex and Gender in Research

Cell line source(s)

HEK293T were originally supplied by the European Collection of Authenticated Cell Cultures (ECACC, Cat No 12022001, Lot No 13D020)

Authentication

For Cell Authentication STR (Short Tandem Repeat) Profiling was performed using the Promega PowerPlex16HS system. This

	profile was compared back to the available profile on commercial cell banks (such as ATCC). The species was confirmedusing a primer system based on the Cytochrome C Oxidase Subunit 1 gene from mitochondria.
Mycoplasma contamination	The specific aliquot of cells used for our experiments was provided by the Cell Services STP of the Francis Crick Institute and was negative for mycoplasma.
Commonly misidentified lines (See ICLAC register)	No commonly misidentified lines were used in this study.

Animals and other research organisms

Policy information about studies involving animals; ARRIVE guidelines recommended for reporting animal research, and Sex and Gender in Research

Laboratory animals	The Sox10CreERT2 transgenic mice refer to two sublines, SER26 (MGI: 5301107) and SER93 (MGI: 5910373) that have been reported previously1-3. Generation of the Foxd3fl allele (MGI: 3790794) and the Wnt1Cre2 transgene have been described previously4,5. The Cre-dependent reporters used are: Rosa26-tdTomato (MGI: 3809524)6 and Rosa26- nuclearGFP (MGI: 5443817)7. Sox10CreER|tdT and Sox10CreER|YFP indicate Sox10CreERT2(SER93);Rosa26-tdTomato and Sox10CreERT2(SER93);Rosa26-YFP mice, respectively. Mice of the desired developmental stage were generated by setting up timed pregnancies. The plug day was designated as E0.5 and date of birth as P0. For adult mice, age-matched mice aged between 2 and 4 months were used for each experiment and cohorts were of mixed genders.
Wild animals	No wild animals included
Reporting on sex	Both male and female animals were used for the experiments.
Field-collected samples	No field-collected samples included
Ethics oversight	All animal procedures were carried out at the Francis Crick Institute in accordance with the regulatory standards of the UK Home Office (ASPA 1986) and the ARRIVE guidelines and approved by the local Animal Welfare and Ethics Review Body (AWERB).

Note that full information on the approval of the study protocol must also be provided in the manuscript.
